# De Novo Assembly of Transcriptome Sequencing in *Caragana korshinskii Kom*. and Characterization of EST-SSR Markers

**DOI:** 10.1371/journal.pone.0115805

**Published:** 2015-01-28

**Authors:** Yan Long, Yanyan Wang, Shanshan Wu, Jiao Wang, Xinjie Tian, Xinwu Pei

**Affiliations:** 1 Institute of Biotechnology, Chinese Academy of Agricultural Sciences, Beijing, 100081, China; 2 College of Plant science and technology, Huazhong Agricultural University, Wuhan, 430070, China; Huazhong university of Science and Technology, CHINA

## Abstract

*Caragana korshinskii Kom*. is widely distributed in various habitats, including gravel desert, clay desert, fixed and semi-fixed sand, and saline land in the Asian and African deserts. To date, no previous genomic information or EST-SSR marker has been reported in *Caragana Fabr*. genus. In this study, more than two billion bases of high-quality sequence of *C. korshinskii* were generated by using illumina sequencing technology and demonstrated the de novo assembly and annotation of genes without prior genome information. These reads were assembled into 86,265 unigenes (mean length = 709 bp). The similarity search indicated that 33,955 and 21,978 unigenes showed significant similarities to known proteins from NCBI non-redundant and Swissprot protein databases, respectively. Among these annotated unigenes, 26,232 a unigenes were separately assigned to Gene Ontology (GO) database. When 22,756 unigenes searched against the Kyoto Encyclopedia of Genes and Genomes Pathway (KEGG) database, 5,598 unigenes were assigned to 5 main categories including 32 KEGG pathways. Among the main KEGG categories, metabolism was the biggest category (2,862, 43.7%), suggesting the active metabolic processes in the desert tree. In addition, a total of 19,150 EST-SSRs were identified from 15,484 unigenes, and the characterizations of EST-SSRs were further compared with other four species in *Fabraceae*. 126 potential marker sites were randomly selected to validate the assembly quality and develop EST-SSR markers. Among the 9 germplasms in *Caranaga Fabr*. genus, PCR success rate were 93.7% and the phylogenic tree was constructed based on the genotypic data. This research generated a substantial fraction of transcriptome sequences, which were very useful resources for gene annotation and discovery, molecular markers development, genome assembly and annotation. The EST-SSR markers identified and developed in this study will facilitate marker-assisted selection breeding.

## Introduction

*Caragana* is a genus comprises about 100 species in the family *Fabraceae*, and distributes in Asia and Eastern Europe. Most of the *Caragana* species are shrubs or small trees and with the character of high tolerance to several abiotic stresses including drought, salt, and cold [1]. However, compared with its high ecological and economic values, the genome and genetic essence remain largely unknown because of little genomic information. *Caragana korshinskii*, which is widely distributes in sandy grassland in northwestern China and Mongolia[2], is a useful model organism for studying salt and drought resistance mechanisms in *Caragana Fabr*. because it can tolerate severe drought stress with 7.38% of soil water content[3]. Much of the work conducted on this species to date has focused on the physiological mechanisms responsible for its resistance to abiotic factors [4,5]. More recent studies using different types of molecular markers, such as RAPD[6], AFLP[1,7] have provided useful information on its genetics and evolutionary history. However, partly because of the scarcity of suitable molecular markers, much remains to be learned about the genetic factors responsible for the ability of *C*. *korshinskii* to cope with various adverse environmental conditions.

Molecular markers play important roles in many aspects of plant breeding, such as genetic diversity research [8], marker-assisted selection [9], and identification of genes that are responsible for desirable traits [10]. SSR is one of the most often used molecular markers and widely used in different aspects of agronomic research [11,12]. Traditional SSR marker development needs partial genomic DNA library construction, cloning and labour-intensive Sanger sequencing [13,14]. With the application of next-generation sequencing (NGS) technology, it has become possible to develop large numbers of SSR markers for non-model organisms quickly and cost-efficiently [15,16]. The transcriptome profile provides information on gene expression and regulation. Therefore, transcriptome analysis is essential to interpret the functional elements of the genome and reveal the molecular components of cells and tissues [17,18]. Transcriptome sequencing is an efficient way to generate functional genomic-level data for non-model organisms. Large collections of EST sequences are very important for gene annotation and discovery [19], comparative genomics [20], development of molecular markers [16], and population genomics studies of genetic variation associated with adaptive traits [21]. Until now, transcriptomic sequencing for SSR mining has been used in a wide range of angiosperm species, such as rubber tree [22], castor bean[23], sesame[24]. Furthermore, transcriptome-derived SSR markers have been found close to or within the functional genes [25,26]. And it was found the characters of di-, tri-, tetra-, penta- and hexa-nucleotide SSRs varying in different taxa. For example, tri-nucleotide repeats have generally been observed to have the highest frequency in many crops, including cotton, barley, wheat, maize, sorghum, rice and peanut [27–29]. While, in sesame and some *Rosaceae* species, the most abundant repeat motif type was the di-nucleotide type [24,30]. Transcriptomic information, however, is extremely lacking for the species of *Caragana Fabr*. Until now, there has been little interest in such data.

Companion with the NGS technology developing, an excellent opportunity exists to explore the issues related to SSR markers from the transcriptome of *C*. *korshinskii*. In this study, we first obtained the transcriptome of *C*. *korshinskii* by Illumina sequencing to validate and characterize microsatellite markers. Based on these databases, thousands of SSR loci were used to design SSR primers. A sample of these primers was further developed to estimate genetic diversity of nine representative species in *Caragana Fabr*.genus.

## Materials and Methods

### Plant materials collection and preparation

*Caragana korshinskii* seeds were provided by the Gansu Desert Control Institute. The seeds were sown on damp filter paper and incubated at 4°C for 4 days before being placed at 23°C under long-day (16 h light/8 h dark) conditions with a photosynthetic photon flux density of 150 μmol m^-2^ s^-1^. After growth for one month, the different tissues from seedlings, including leaves, stems and roots, were harvested for RNA isolation.

### RNA isolation and transcriptome sequencing

The total RNA of plants was extracted with TRIzol Reagent (Invitrogen, 15596–026) according to the manufacturer’s instructions. The RNA samples that met the requirements were used to construct transcriptome sequence libraries. The total RNA of each sample was then pooled at equivalent quantities. Sequencing libraries were generated using a NEBNext Ultra RNA Library Prep Kit for Illumina (NEB, USA) following the manufacturer’s recommendations. Following the manufacturer’s procedures, mRNA was purified from the pooled total RNA using polyT oligo-attached magnetic beads. A fragmentation buffer was added to disrupt the mRNA into short fragments. Reverse transcriptase and random primers were used to synthesise the first-strand cDNA from the cleaved mRNA fragments. The second-strand cDNA was synthesised using buffer, dNTPs, RNaseH, and DNA polymeraseI. The double-strand cDNA was purified using the QIAquick PCR extraction kit (QIAGEN, Hilden, Germany) and washed with EB buffer for end repair and single nucleotide A (adenine) addition. Finally, sequencing adaptors were ligated onto the fragments. The required fragments were purified by AMPure XP beads and enriched by PCR to construct a library for transcriptome sequencing.

### Data filtering and de novo assembly

The transcriptome library was sequenced using the Illumina HiSeq 2000 system. The sequencing-received raw image data were transformed by base calling into the sequence data, which were termed raw reads. The raw data were then filtered by data-processing steps to generate clean data via a process that included the removal of adapter sequences, reads in which unknown bases are greater than 10%, and low-quality sequences (the percentage of low-quality bases of quality value ≤5 is greater than 50% in a read). All of the raw data was submitted to the database with the code Bioproject: SUB718537 and BioSample: SAMN03121496. After obtaining the clean data, transcriptome assembly was accomplished by using Trinity software [31] with min_kmer_cov set to 2 by default and all other parameters set at default values.

### Functional annotation of unigenes

For functional annotation, the assembled unigenes that might putatively encode proteins were searched against NR (http://www.ncbi.nlm.nih.gov/), Swiss-Prot (http://www.expasy.ch/sprot/), KEGG (http://www.genome.jp/kegg/) using the BLASTX algorithm. A typical cut-off value of E<1e-5 was used. With the NR annotation, the Blast2GO program [32] was used to obtain the GO annotation of unigenes according to component function, biological process and cellular component ontologies. After obtaining a GO annotation for every unigene, WEGO software[33] was used to perform GO functional classification for all unigenes and to understand the distribution of gene functions of the species at the macro level.

### SSR mining and primer design

The RNA-seq data from the other four *Fabrceae* species, *Cicer arietium*, *Lotus corniculatus*, *Medicago sativa*, and *Medicago truncatula* were got from the database PlantGDB(http://www.plantgdb.org/). Then, the MISA software (http://pgrc.ipk-gatersleben.de/misa/misa.html) was used to identify microsatellites in the unigenes got in this study and the four above database. The standard of EST-SSRs was assumed to contain motifs of one to six nucleotides in size. Definement of microsatellites was used with the following settings, SSR repeat motifs and number of repeats shown respectively, mono-10, dimer-6, trimer-5, tetramer-5, pentamer-5, hexamer-5.The primer for each SSR was designed using Primer3 (http://primer3.sourceforge.net/releases.php).

### PCR amplification and validation of selected EST-SSR markers

All primer pairs were screened for amplification and polymorphisms using DNA from 9 *Caragana* species including *C.opulens Kom*., *C*.*microphylla Lam*., *C*.*intermedia Kuang et H*.*C*.*Fu*, *C*.*arborescens Lam*., *C*.*rosea Turcz*.*ex Maxim*, *C*.*roborovskyi Kom*., *C*.*stenophylla Pojark*., *C*.*acanthophylla Kom*. *C*.*korshinskii Kom*. In total, 126 pairs of primers were designed (S3 Table) and validated by PCR. The DNA for PCR amplification was extracted from the control samples using the CTAB method[34]. PCR amplification was carried out as follows: 94°C for 4 min, followed by 35–40 cycles of 94°C for 30 s, 55–60°C for 30 s and 72°C for 30 s. The final extension was performed at 72°C for 10 min. The PCR products were analysed by electrophoresis on 1.0% agarose gels. Coefficients of genetic similarity for the 9 species used in this study were calculated using the SIMQUAL program of NTSYS-pc Version 2.10 [35]. A neighbor-joining dendrogram was constructed based on the genetic similarity matrix with the SHAN clustering program of NTSYS-pc using the UPGMA algorithm.

## Results

### Illumina paired-end sequencing and de novo assembly

To elucidate the transcriptome of *C*. *korshinskii*, RNA was extracted from different tissues and sequenced with Illumina paired-end sequencing technology. In this study, a total of 66,351,948 raw sequencing reads with a length of 100 bp were generated from a 200 bp insert library. After removing adaptors and low-quality data, 64,031,599 clean reads were obtained. Then, the high-quality reads were used to assemble the transcriptome data with Trinity software. According to the overlapping information of high-quality reads, 202,163 transcripts were generated with an average length of 1,089 bp and an N50 of 1,772 bp. After extracting the longest transcript for each transcript, 86,265 unigenes were obtained. The average length was 709 bp, and the length greater than 500 bp accounted for approximately 37.27% (Table 1, S1 Fig.).

**Table 1:** Summary of the *Caragana korshinskii* transcriptome.

Category	Number				Total number	Mean length (bp)	N50 (bp)	Total nucleotides
	200–500bp	500–1kbp	1k-2kbp	>2kbp				
Transcripts	78,801	44,253	47,654	31,455	202,163	1,089	1772	220,191,379
Unigenes	54,117	15,417	10,235	6496	86,265	709	1231	61,128,411

### Annotation of all nonredundant unigenes

For the validation and annotation of the assembled unigenes, the assembled unigenes were searched against the NCBI non-redundant (NR) and SwissProt protein databases using the BLAST2 program with an E-value threshold of 1e-5. Among 86,265 unigenes, 33,955 (39.36%) had significant similarity to 21,118 unique proteins. Of all of the unigenes, 21,978 (25.47%) with significant identities to SwissProt proteins were matched with 21,978 unique protein accessions (Table 2). A lower percentage was obtained when searching against the SwissProt protein database. In total, BLAST searches identified 16,533 unique protein accessions from the NR and SwissProt protein databases, suggesting that this Illumina paired-end sequencing project generated a substantial fraction of the expressing genes in this study.

**Table 2:** Summary of functional annotation of assembled unigenes.

Public database	Number of Unigenes	Percentage (%)
Annotated in NR	33,955	39.36
Annotated in NT	28,400	32.92
Annotated in KO	5598	6.48
Annotated in SwissProt	21,978	25.47
Annotated in PFAM	22,956	26.61
Annotated in GO	26,232	30.4
Annotated in KOG	10,867	12.59
Annotated in all Databases	3012	3.49
Annotated in at least one Database	41,493	48.09
Total Unigenes	86,265	100

### Functional classification by GO analysis

**Fig 1:**
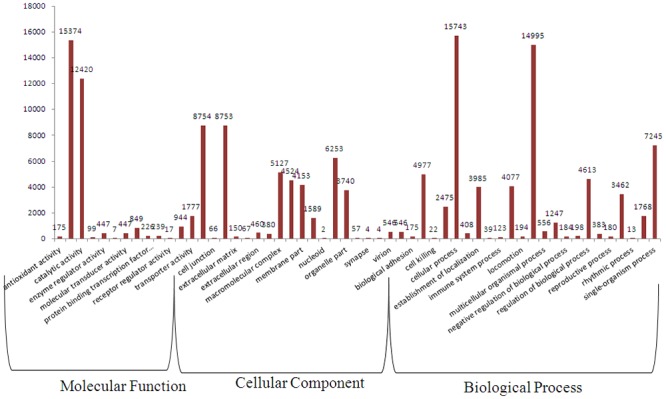
Functional classification of assembled unigenes. Functional classification of assembled unigenes based on Gene Ontology (GO) categorisation. The results are summarised in three main GO categories: biological process, cellular component and molecular function. The x-axis indicates the subcategories, and the y-axis indicates the numbers related to the total number of GO terms present; the unigene numbers that are assigned the same GO terms are indicated on the top of the bars.

Gene ontology (GO), is an internationally standardised gene functional classification system. In order to classify the functions of the predicted *C*. *korshinskii* unigenes, GO analysis was performed. In total, 26,232 unigenes with BLAST matches to known proteins were assigned to GO classes with 9787 functional terms (Table 2, S1 Table). As shown in Fig. 1, assignments to the biological process constituted the majority (67,062, 46.17%), followed by cellular component (45,175, 31.1%) and molecular function (33,021, 22.73%).

Under the category of biological process, cellular process (15,743, 23.48%) and metabolic process (14995, 22.36%) were prominent, indicating that important cell processes and metabolic activities occurred in *C*. *korshinskii*. Under the classification of molecular function, binding (15,374, 46.6%) and catalytic activity (12,420, 37.6%) were the first and second largest categories, respectively, whereas other categories, such as transporter activity, structural molecule activity, nucleic acid binding transcription factor activity, and molecular transducer activity, contained 4017 unigenes, representing only 12.17%. Regarding the cellular components, two categories—cell and cell part—represented approximately 38.75% of cellular components, organelle accounted for approximately 13.84%, and membrane and membrane part accounted for 19.21%.

### Functional classification by the KEGG pathway

**Fig 2:**
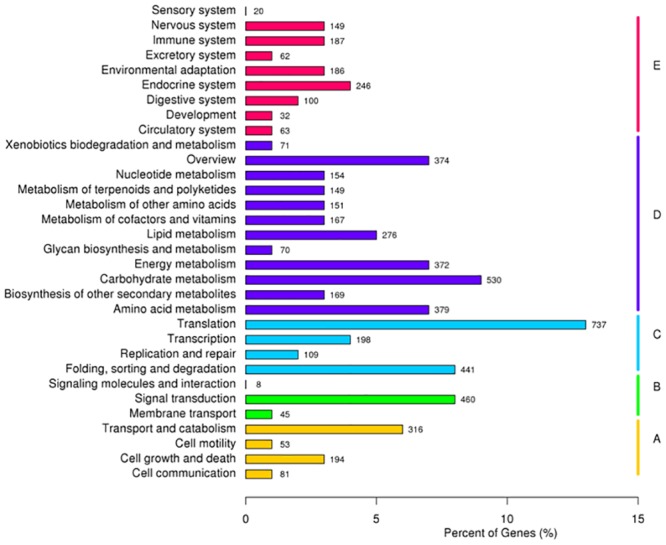
Pathway assignment based on the Kyoto Encyclopedia of Genes and Genomes (KEGG). (A) Classification based on cellular process categories, (B) classification based on environmental information processing categories, (C) classification based on genetic information processing categories, (D) classification based on metabolism categories, and (E) classification based on organismal systems categories.

To further analyse the transcriptome of *C*. *korshinskii*, all of the unigenes were analysed in the KEGG pathway database.The KEGG pathway database is a knowledge base for the systematic analysis of gene functions in terms of networks of genes and molecules in cells and their variants specific to particular organisms. Out of the 86,265 unigenes, 5598 (6.49%) with significant matches in the database were assigned to 5 main categories, including 32 KEGG pathways (Fig. 2, S2 Table). Among these 5 main categories, metabolism was the largest (2862, 43.7%), followed by genetic information (1485, 22.68%), organismal systems (1045, 15.96%), cellular processes (644, 9.83%) and environmental information processing (513, 7.83%). These results indicate that active metabolic processes were on-going. As shown in S2 Table, KEGG metabolism contained 12 categories, such as carbohydrate metabolism, nucleotide metabolism, the biosynthesis of other secondary metabolisms, amino acid metabolism, lipid metabolism, and energy metabolism, among others.

### Motif comparison of EST-SSR markers among 4 *Caragana Fabr*. species

In this study, the 86,265 unigenes generated in this study were used to mine potential microsatellites that were defined as mono- to hexa-nucleotide motifs with a minimum of three repetitions. Using the MISA software, a total of 19,150 potential simple sequence repeats (SSR) were identified in 15,484 unigenes. Of the 15,484 unigenes, 12,575 and 2,909 unigenes contained one and more than one SSR, respectively (Table 3). The number of potential EST-SSR per unigene varied from 1 to 8, with an average of 1.17.

**Table 3:** Summary of the EST-SSRs that were identified in the transcriptome.

Search item	Numbers
Total number of examined unigenes	86,265
Total size of examined sequences (bp)	61,128,411
Total number of identified EST-SSRs	19,150
Number of EST-SSRs containing sequences	15,484
Number of sequences containing more than one EST-SSR	2909
Mono-nucleotide	11,472
Di-nucleotide	3924
Tri-nucleotide	3433
Tetra-nucleotide	284
Penta-nucleotide	26
Hexa-nucleotide	11

**Fig 3:**
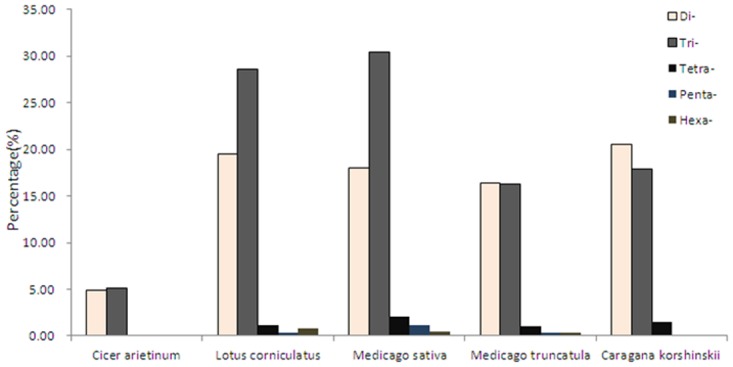
SSRs motif distribution analysis in *Caragana korshinskii Kom*. and 4 supplementary materials from public database. The bars from left to right in each species represent di-, tri-, tetra-, penta- and hexa-nucleotides.

To obtain a comprehensive perspective of motif distribution, we further compared our results with data from other species in *Fabraceae*. Besides Mono-nucleotide type, the di- and tri- type were the most two frequent types (Fig. 3). Comparing with other 4 species, the di-nucleotide was the most abundant type, while for the other 4 species, the tri-nucleotide was the most frequent type. The dominant di-nucleotide repeat motif in SSRs was AG/CT, whereas CG/GC was the least abundant (Table 4). Among the tri-nucleotide repeats, the most frequent repeat was AAG/CTT, followed by ACC/GTT (19.6%) and AAT/ATT. The most frequent of the motif was consistent with the other 4 species. While in *Lotus corniculatus*, ACC/GGT motif was the second abundant type, and in *Medicago sativa* and *Medicago truncatula*, the third abundant type was the ACC/GGT motif.

**Table 4:** Comparasion of three types of motifs for EST-SSR in all the five species.

Motif	Cicer arietium	Lotus corniculatus	Medicago sativa	Medicago truncatula	Caragana korshinskii
A/T	5445(75)*	2126(46.5)	442(47.1)	9061(70.5)	11150(58.22)
C/G	1052(14.5)	138(3.02)	8(0.85)	785(5.23)	322(1.68)
AG/CT	216(3)	575(12.6)	119(12.7)	1766(17.8)	2584(13.5)
AT/AT	108(1.5)	204(4.5)	19(2.0)	468(3.1)	460(2.4)
AC/GT	30(0.4)	112(2.5)	31(3.3)	201(1.3)	873(4.6)
CG/CG	3(0.04)	2(0.04)	-	31(0.2)	7(0.04)
AAG/CTT	96(1.4)	414(9.1)	97(10.3)	851(5.7)	1035(5.4)
AAC/GTT	55(0.8)	143(3.1)	40(4.3)	313(2.1)	619(3.2)
AAT/ATT	69(0.95)	49(1.1)	23(2.5)	297(2.0)	360(1.9)
ACC/GGT	47(0.6)	246(5.4)	39(4.2)	211(1.4)	326(1.7)
ACG/CGT	4(0.06)	16(0.4)	2(0.2)	35(0.2)	40(0.2)
ACT/AGT	9(0.1)	32(0.7)	2(0.2)	74(0.5)	114(0.6)
AGC/CTG	15(0.2)	79(1.7)	26(2.8)	141(0.9)	181(0.9)
AGG/CCT	16(0.2)	119(2.6)	12(1.3)	119(0.8)	295(1.5)
ATC/ATG	59(0.8)	166(3.6)	43(4.6)	391(2.6)	395(2.1)
CCG/CGG	4(0.06)	44(1.0)	1(0.1)	18(0.1)	68(0.4)

*： The numbers in the bracket showed the percentage of the specific SSR motif type.

### Validation of EST-SSR markers

Based on the SSR-containing sequences, 126 SSR sites were randomly selected to design EST-SSR primers with Primer Premier 3.0. The information of the EST-SSR primers is shown in S3 Table. Among the 126 primer pairs, 118 were successful in PCR amplification with genomic DNA, and the remaining eight pairs of primers failed to generate PCR products at various annealing temperatures. Of the 118 working primer pairs, 98 PCR products showed specific amplification, among which 90 PCR products generated expected sizes, whereas the other nine generated PCR products that were larger than expected, suggesting that the amplified regions likely contained introns. A total of 20 PCR products generated more than one band, which might result from the primer design or the high heterozygosity of the Caragana germplasm.

**Fig 4:**
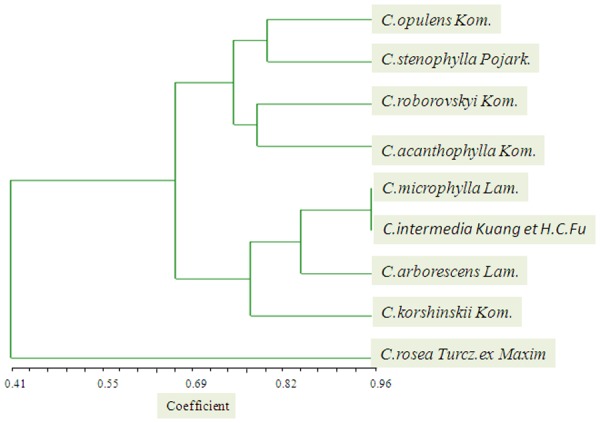
Graph of genetic distance among the UPGMA dendrogram of the genetic relationships among 9 species from *Caragana Fabr* genus. The dendrogram was generated using the Jaccard similarity coefficient based on 118 polymorphic primer pairs.

All polymorphic loci were used to analyse the diversity of 9 species. The observed number of alleles (A) ranged from 1 to 5, with an average of 2.12 alleles per locus. The genetic distance was calculated by the NTSYS software. It was showed that for the 9 species could be divided into two groups (Fig. 4), the *C*.*rosea Turcz*.*ex*.*Maxim* was the far distance with the other species. The other eight species could be divided into two groups. Among the groups, the *C*.*Korshinskii* was closet to *C*.*microphylla*, *C*.*intermedia Kuang et H*.*C*.*Hu* and *C*.*arborescens Lam*.

## Discussion

In this study, a large number of *C*.*Korshinskii* transcriptomic unigenes (86,265) were sequenced with the Illumina HiSeq 2000 platform (Table 1). The N50 length of the unigenes was 1231 bp, and the average length was 709bp.These results are comparable to recently published plant transcriptomic analyses, such as that of *Gossypium aridum* (N50 = 593 bp) [36] and *Momordica cochinchinensis*(N50 = 450bp)[37]. Trinity is one of most powerful packages in the de novo assembly of short reads. In this study, fewer than half of the unigenes (41,493, 48.09%) were successfully annotated by BLAST against the public databases Nr, Nt, Swiss-Prot, GO and KEGG, given the absence of genomic information of *C*. *Korshinskii* (Table 2). Notably, the percentage of annotation is relative low among the previous studies using the same sequencing strategy during the last year (55 to 78.9% [38–40]). One possible reason of this lack of annotation is technical limitations, such as sequencing depth and read length[41], which were common in all of the studies using de novo transcriptome analysis. The unannotated sequences were, on average, much shorter than were the annotated unigenes (382 bp vs 1182 bp).

The EST-SSR marker is important for a variety of research, including the assessment of genetic diversity, the development of genetic maps, comparative genomics, marker-assisted selection, and other fields. Until now, there has been no report of EST-SSR identification in desert trees. The transcriptome sequencing provided many sequences for developing numerous EST-SSR markers in the *C*.*Korshinskii* tree. In total, 19,150 potential EST-SSRs were identified from 15,484 unigenes. In this study, in addition to the common di-, tri- and other nucleotide repeats that were included in the selection, the mono-nucleotide repeats included SSR, and its proportion was greater than that of the other types. If mono-nucleotide repeats were excluded, the frequency of di-nucleotide was higher than that of tri-nucleotide in *C*.*Korshinskii*, which is different with the other 4 species. In previous studies, some of the results showed that di-nucleotide was the most abundant type, such as sesame[24], oilpalm[42], while other results showed tri-nucleotide was the most abundant type, like barley[43], wheat[29]. The most abundant di- and tri-nucleotide motifs were AG/TC and AAG/TTC, respectively. These results are consistent with results for dicots, such as oak [44] and castor bean [23].

Of 126 primer pairs that were randomly selected for PCR validation, 118 (93.7%) produced clear bands. The PCR success rate was the same as in previous studies, such as *Populus euphratica* [45], and higher than the results from Triwitayakorn et al. (75%) [46], which mean that the identified EST-SSR makers have high cross-transferability in *Caragana* genus. The polymorphism frequency among the 9 species was 90.5%, and this ratio is much higher than that in crops. For example, in sesame, 276 (92.0%) EST-SSR primer pairs yielded PCR amplification products in 24 cultivars. Thirty two primer pairs (11.59%) exhibited polymorphisms. Moreover, 203 primer pairs (67.67%) yielded PCR amplicons in the wild accession and 167 (60.51%) were polymorphic between species[24]. In peanut, 26 (10.3%) EST-SSRs exhibited polymorphisms between 22 cultivated peanut accessions and 221 (88%) were polymorphic between 16 wild peanut species[28].

Our dendrogram, based on genetic similarity results, divided the 9 species into 2 clear groupings. Among the group, the *C*.*Korshinskii* was closet to *C*.*microphylla*, *C*.*intermedia Kuang et H*.*C*.*Hu* and *C*.*arborescens Lam*. This result was consistent with other previous researches[47]. While for the other group, the *C*.*roborovskyi Kom*. was classified into the group, this result was different with the classification described by Zhao (1993)[47,48]. He divided the *Caragana Fabr*. genus into three sub-groups, and *C*.*roborovskyi Kom*. was the only one species in one sub-group. While the classification was only based on morphology investigation, it needed more molecular evidence to support. So the result in our research extended the researches for *Caragana Fabr*. *genus*.

## Conclusion

To sum up, our data provided a case study that the microsatellite markers developed from transcriptome of *C*. *korshinskii Kom*. can be used for population genetic studies in *Caranaga Fabr*. Likewise, these markers should be of great value in further research on population and conservation genetics of species in the genus. The use of transcriptome sequences from next-generation sequencing, being rapid and efficient in the development of microsatellite markers, is of great value, not only for analysing intraspecies genetic diversity, but for future research across the genus.

## Supporting Information

S1 Fig Length distribution of assembly transcripts and unigenes. (TIF)

S1 Table Summary of the GO classification of assembled unigenes. (XLSX)

S2 Table Summary of the KEGG classification of assembled unigenes. (XLSX)

S3 Table Primer information for EST-SSR markers. (XLSX)

## References

[1] Wang Z, Gao HW, Wu YQ, Han JG (2007) Genetic diversity and population structure of Caragana korshinskii revealed by AFLP. Crop Science 47: 1737–1743.

[2] Wang Z, Gao H (2008) Progress on genetic diversity of Genus Caragana germplasm resources. Journal of Plant Genetic Resources 9(3): 397–400.

[3] Yj Li, Zhao Z Sun Dx, Han G (2008) Hydrological physiological characteristics of Caragana korshinskii under water stress. Journal of Northwest Forestry University 23(3): 1–4.

[4] Yang DH, Song LY, Hu J, Yin WB, Li ZG, et al (2012) Enhanced tolerance to NaCl and LiCl stresses by over-expressing Caragana korshinskii sodium/proton exchanger 1 (CkNHX1) and the hydrophilic C terminus is required for the activity of CkNHX1 in Atsos3–1 mutant and yeast. Biochem Biophys Res Commun 417: 732–737. 10.1016/j.bbrc.2011.12.023 22197553

[5] Wang X, Chen X, Liu Y, Gao H, Wang Z, et al (2011) CkDREB gene in Caragana korshinskii is involved in the regulation of stress response to multiple abiotic stresses as an AP2/EREBP transcription factor. Mol Biol Rep 38: 2801–2811. 10.1007/s11033-010-0425-3 21127996

[6] Ma CC, Gao YB, Liu HF, Wang JL, Guo HY (2003) Interspecific transition among Caragana microphylla, C-davazamcii and C-korshinskii along geographic gradient. I. Ecological and RAPD evidence. Acta Botanica Sinica 45: 1218–1227.

[7] Zhang M, Fritsch PW, Cruz BC (2009) Phylogeny of Caragana (Fabaceae) based on DNA sequence data from rbcL, trnS—trnG, and ITS. Molecular Phylogenetics and Evolution 50: 547–559. 10.1016/j.ympev.2008.12.001 19100848

[8] Naegele R, Tomlinson AJ, Hausbeck MK (2014) Evaluation of a diverse, worldwide collection of wild, cultivated and landrace peppers (Capsicum annuum) for resistance to Phytophthora fruit rot, genetic diversity and population structure. Phytopathology. 10.1094/PHYTO-02-14-0031-R 25054617

[9] Ahmad Z, Mumtaz AS, Ghafoor A, Ali A, Nisar M (2014) Marker Assisted Selection (MAS) for chickpea Fusarium oxysporum wilt resistant genotypes using PCR based molecular markers. Mol Biol Rep 41: 6755–6762. 10.1007/s11033-014-3561-3 25017202 PMC4173118

[10] Talukder ZI, Gong L, Hulke BS, Pegadaraju V, Song Q, et al (2014) A High-Density SNP Map of Sunflower Derived from RAD-Sequencing Facilitating Fine-Mapping of the Rust Resistance Gene R12. PLoS One 9: e98628 10.1371/journal.pone.0098628 25014030 PMC4094432

[11] Miah G, Rafii MY, Ismail MR, Puteh AB, Rahim HA, et al (2013) A Review of Microsatellite Markers and Their Applications in Rice Breeding Programs to Improve Blast Disease Resistance. International Journal of Molecular Sciences 14: 22499–22528. 10.3390/ijms141122499 24240810 PMC3856076

[12] Cuadrado A, Cardoso M, Jouve N (2008) Physical organisation of simple sequence repeats (SSRs) in Triticeae: structural, functional and evolutionary implications. Cytogenet Genome Res 120: 210–219. 10.1159/000121069 18504349

[13] Varshney RK, Marcel TC, Ramsay L, Russell J, Roder MS, et al (2007) A high density barley microsatellite consensus map with 775 SSR loci. Theoretical and Applied Genetics 114: 1091–1103. 17345060 10.1007/s00122-007-0503-7

[14] Nunome T, Negoro S, Kono I, Kanamori H, Miyatake K, et al (2009) Development of SSR markers derived from SSR-enriched genomic library of eggplant (Solanum melongena L.). Theor Appl Genet 119: 1143–1153. 10.1007/s00122-009-1116-0 19662343

[15] Zalapa JE, Cuevas H, Zhu HY, Steffan S, Senalik D, et al (2012) Using Next-Generation Sequencing Approaches to Isolate Simple Sequence Repeat (Ssr) Loci in the Plant Sciences. American Journal of Botany 99: 193–208. 10.3732/ajb.1100394 22186186

[16] Wang BH, Zhu P, Yuan YL, Wang CB, Yu CM, et al (2014) Development of EST-SSR markers related to salt tolerance and their application in genetic diversity and evolution analysis in Gossypium. Genetics and Molecular Research 13: 3732–3746. 10.4238/2014.May.13.1 24854659

[17] Zhou A, Pawlowski WP (2014) Regulation of meiotic gene expression in plants. Front Plant Sci 5: 413 10.3389/fpls.2014.00413 25202317 PMC4142721

[18] Yan D, Duermeyer L, Leoveanu C, Nambara E (2014) The Functions of the Endosperm During Seed Germination. Plant Cell Physiol 55: 1521–1533. 10.1093/pcp/pcu089 24964910

[19] Emrich SJ, Barbazuk WB, Li L, Schnable PS (2007) Gene discovery and annotation using LCM-454 transcriptome sequencing. Genome Research 17: 69–73. 17095711 10.1101/gr.5145806 PMC1716268

[20] Vera JC, Wheat CW, Fescemyer HW, Frilander MJ, Crawford DL, et al (2008) Rapid transcriptome characterization for a nonmodel organism using 454 pyrosequencing. Molecular Ecology 17: 1636–1647. 10.1111/j.1365-294X.2008.03666.x 18266620

[21] Namroud MC, Beaulieu J, Juge N, Laroche J, Bousquet J (2008) Scanning the genome for gene single nucleotide polymorphisms involved in adaptive population differentiation in white spruce. Mol Ecol 17: 3599–3613. 10.1111/j.1365-294X.2008.03840.x 18662225 PMC2613251

[22] Li DJ, Deng Z, Qin B, Liu XH, Men ZH (2012) De novo assembly and characterization of bark transcriptome using Illumina sequencing and development of EST-SSR markers in rubber tree (Hevea brasiliensis Muell. Arg.). Bmc Genomics 13 10.1186/1471-2164-13-591 22607098 PMC3431226

[23] Qiu L, Yang C, Tian B, Yang J-B, Liu A (2010) Exploiting EST databases for the development and characterization of EST-SSR markers in castor bean (Ricinus communis L.). BMC Plant Biology 10: 278 10.1186/1471-2229-10-278 21162723 PMC3017068

[24] Zhang HY, Wei LB, Miao HM, Zhang TD, Wang CY (2012) Development and validation of genic-SSR markers in sesame by RNA-seq. Bmc Genomics 13 10.1186/1471-2164-13-591 22800194 PMC3428654

[25] Li YC, Korol AB, Fahima T, Beiles A, Nevo E (2002) Microsatellites: genomic distribution, putative functions and mutational mechanisms: a review. Molecular Ecology 11: 2453–2465. 12453231 10.1046/j.1365-294x.2002.01643.x

[26] Morgante M, Hanafey M, Powell W (2002) Microsatellites are preferentially associated with nonrepetitive DNA in plant genomes. Nature Genetics 30: 194–200. 11799393 10.1038/ng822

[27] Wang CB, Guo WZ, Cai CP, Zhang TZ (2006) Characterization, development and exploitation of EST-derived microsatellites in Gossypium raimondii Ulbrich. Chinese Science Bulletin 51: 557–561.

[28] Liang X, Chen X, Hong Y, Liu H, Zhou G, et al (2009) Utility of EST-derived SSR in cultivated peanut (Arachis hypogaea L.) and Arachis wild species. BMC Plant Biology 9: 35 10.1186/1471-2229-9-35 19309524 PMC2678122

[29] Kantety RV, La Rota M, Matthews DE, Sorrells ME (2002) Data mining for simple sequence repeats in expressed sequence tags from barley, maize, rice, sorghum and wheat. Plant Mol Biol 48: 501–510. 11999831 10.1023/a:1014875206165

[30] Jung S, Abbott A, Jesudurai C, Tomkins J, Main D (2005) Frequency, type, distribution and annotation of simple sequence repeats in Rosaceae ESTs. Functional Integrative Genomics 5: 136–143. 15761705 10.1007/s10142-005-0139-0

[31] Grabherr MG, Haas BJ, Yassour M, Levin JZ, Thompson DA, et al (2011) Full-length transcriptome assembly from RNA-Seq data without a reference genome. Nature Biotechnology 29: 644–U130. 10.1038/nbt.1883 21572440 PMC3571712

[32] Conesa A, Gotz S, Garcia-Gomez JM, Terol J, Talon M, et al (2005) Blast2GO: a universal tool for annotation, visualization and analysis in functional genomics research. Bioinformatics 21: 3674–3676. 16081474 10.1093/bioinformatics/bti610

[33] Ye J, Fang L, Zheng H, Zhang Y, Chen J, et al (2006) WEGO: a web tool for plotting GO annotations. Nucleic Acids Res 34: W293–297. 16845012 10.1093/nar/gkl031 PMC1538768

[34] Del Sal G, Manfioletti G, Schneider C (1989) The CTAB-DNA precipitation method: a common mini-scale preparation of template DNA from phagemids, phages or plasmids suitable for sequencing. Biotechniques 7: 514–520. 2699240

[35] Mogea JP (1999) Relationships and phylogeny of the species of the genus Arenga (Palmae) based on morphology using the polarity method and the NTSYS program. Evolution, Variation, and Classification of Palms 83: 169–177.

[36] Xu P, Liu Z, Fan X, Gao J, Zhang X, et al (2013) De novo transcriptome sequencing and comparative analysis of differentially expressed genes in Gossypium aridum under salt stress. Gene 525: 26–34. 10.1016/j.gene.2013.04.066 23651590

[37] Hyun TK, Rim Y, Jang HJ, Kim CH, Park J, et al (2012) De novo transcriptome sequencing of Momordica cochinchinensis to identify genes involved in the carotenoid biosynthesis. Plant Molecular Biology 79: 413–427. 10.1007/s11103-012-9919-9 22580955

[38] Liu M, Qiao G, Jiang J, Yang H, Xie L, et al (2012) Transcriptome sequencing and de novo analysis for Ma bamboo (Dendrocalamus latiflorus Munro) using the Illumina platform. PLoS One 7: e46766 10.1371/journal.pone.0046766 23056442 PMC3463524

[39] Lulin H, Xiao Y, Pei S, Wen T, Shangqin H (2012) The first Illumina-based de novo transcriptome sequencing and analysis of safflower flowers. PLoS One 7: e38653 10.1371/journal.pone.0038653 22723874 PMC3378585

[40] Xu DL, Long H, Liang JJ, Zhang J, Chen X, et al (2012) De novo assembly and characterization of the root transcriptome of Aegilops variabilis during an interaction with the cereal cyst nematode. BMC Genomics 13: 133 10.1186/1471-2164-13-133 22494814 PMC3439707

[41] Novaes E, Drost DR, Farmerie WG, Pappas GJ Jr, Grattapaglia D, et al (2008) High-throughput gene and SNP discovery in Eucalyptus grandis, an uncharacterized genome. BMC Genomics 9: 312 10.1186/1471-2164-9-312 18590545 PMC2483731

[42] Ting NC, Zaki NM, Rosli R, Low ET, Ithnin M, et al (2010) SSR mining in oil palm EST database: application in oil palm germplasm diversity studies. J Genet 89: 135–145. 20861564 10.1007/s12041-010-0053-7

[43] La Rota M, Kantety RV, Yu JK, Sorrells ME (2005) Nonrandom distribution and frequencies of genomic and EST-derived microsatellite markers in rice, wheat, and barley. Bmc Genomics 6 15720707 10.1186/1471-2164-6-23 PMC550658

[44] Durand J, Bodenes C, Chancerel E, Frigerio J-M, Vendramin G, et al (2010) A fast and cost-effective approach to develop and map EST-SSR markers: oak as a case study. BMC Genomics 11: 570 10.1186/1471-2164-11-570 20950475 PMC3091719

[45] Mortazavi A, Williams BA, Mccue K, Schaeffer L, Wold B (2008) Mapping and quantifying mammalian transcriptomes by RNA-Seq. Nature Methods 5: 621–628. 10.1038/nmeth.1226 18516045 PMC13303166

[46] Triwitayakorn K, Chatkulkawin P, Kanjanawattanawong S, Sraphet S, Yoocha T, et al (2011) Transcriptome Sequencing of Hevea brasiliensis for Development of Microsatellite Markers and Construction of a Genetic Linkage Map. DNA Research 18: 471–482. 10.1093/dnares/dsr034 22086998 PMC3223080

[47] Guo Q, Shi Y, Wei Z, Yang Z, Lu J, et al (2008) Genetic diversity analysis by SSR marker of fourteen species of Caragana Fabr. in He-Xi corridor area of Gansu. Acta Agrestia Sinica 16: 227–233.

[48] Kang H, Bai J, Chen K, Wang G (2011) Phylogenetic relationships of Caragana(Fabaceae): evidence from nrITS sequences. Southwest China Journal of Agricultural Sciences 24: 1099–1103.

