# Supplementary figures and images for: De Novo Assembly of Transcriptome Sequencing in Caragana korshinskii Kom. and Characterization of EST-SSR Markers
Source: PLoS One. 2015 Jan 28;10(1):e0115805. doi: 10.1371/journal.pone.0115805 (PMC4309406; doi:10.1371/journal.pone.0115805)

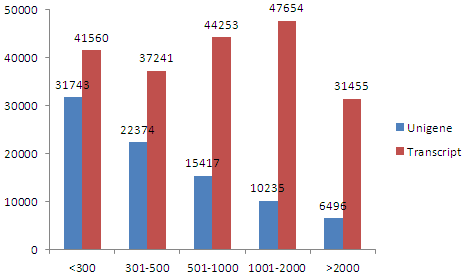

Supplement: S1 Fig — (TIF) [file pone.0115805.s001.tif]
